# Construction of N/S CQDs@Fe-TCPP Nanocatalyst-Induced Electrochemical Sensors for Rapid and Sensitive Detection of Enrofloxacin Residues in Milk

**DOI:** 10.3390/foods15020266

**Published:** 2026-01-11

**Authors:** Wenjing Wang, Shujuan Chen, Yifan Fu, Yike Hong, Chenbo Tang, Likou Zou, Junni Tang, Li He, Shuliang Liu, Kaidi Hu, Aiping Liu

**Affiliations:** 1 College of Food Science, Sichuan Agricultural University, Ya’an 625000, China; w27408222392024@163.com (W.W.); kun20240911@163.com (Y.F.); hhyk2023@163.com (Y.H.); 18096226998@163.com (C.T.); helifood@sicau.edu.cn (L.H.); lsliang999@163.com (S.L.); kaidiminer@163.com (K.H.); aipliu@sicau.edu.cn (A.L.); 2 College of Resources, Sichuan Agricultural University, Chengdu 611130, China; zoulikou@sicau.edu.cn; 3 College of Pharmacy and Food, Southwest Minzu University, Chengdu 610225, China; junneytang@swun.edu.cn

**Keywords:** enrofloxacin, carbon quantum dots, metal–organic framework, complex carbon-based nanozymes, electrochemical sensor

## Abstract

Given the potential hazards of enrofloxacin (ENR) residues to human health, establishing an accurate, rapid, and stable detection method is of importance. To enable the direct detection of ENR, an electrochemical sensor was constructed in this study. N- and S-doped carbon quantum dots (CQDs) with peroxidase-like activity were prepared using DL-malic acid, L-alanine, and L-cysteine as precursors and compounded with a tetrakis (4-carboxyphenyl) porphyrin (TCPP) and Fe(NO_3_)_3_·9H_2_O to make novel N/S CQDs@Fe-TCPP composite carbon-based nanozymes to construct an electrochemical sensor, and the electrochemical behavior was investigated. Under optimal experimental conditions, the sensor exhibited a linear current response to ENR concentrations in the range of 1–1300 nM (I (μA) = 0.0106c (nM) + 2.9861, R^2^ = 0.9962), with a calculated detection limit of 0.872 nM (S/N = 3). The recovery rate of this sensor in actual milk samples ranged from 99.02% to 100.9%. The reproducibility experiments demonstrated the high precision of the method, with a relative standard deviation (RSD) of 1.27%. Stability testing revealed a peak current retention rate of 93.51% on day 21, indicating excellent system stability. These findings indicate that the sensor shows great capability for ENR detection in food products.

## 1. Introduction

Enrofloxacin (ENR) is a third-generation fluoroquinolone antibiotic agent with low resistance crossover, highly effectiveness, and low toxicity, which is broadly used in the therapy of animal and plant diseases owing to its significant antibacterial activity against diverse bacterial pathogens, including both Gram-positive and Gram-negative bacteria [1]. However, inappropriate use of ENR may lead to residues in food exceeding safety standards, which may result in a variety of negative effects on human beings through the enrichment effect of the food chain, including skin allergic reactions, dysbiosis of intestinal microflora, carcinogenic mutations, etc., as well as possible pollution of the environment [2]. China National Food Safety Standard (GB 31650-2019) [3] has stipulated that the maximum residue limit for ENR in milk, chicken, fish, and pork is 100 μg/kg. However, illegal overuse of such drugs persists. Therefore, developing highly efficient and sensitive detection methods for ENR residues in food is vital for protecting human health, creating a persistent demand for practical and reliable monitoring tools.

At present, detecting ENR residues in food products mainly includes high-performance liquid chromatography (HPLC), HPLC coupled with mass spectrometry (LC-MS), enzyme-linked immunosorbent assay (ELISA), microbial detection methods [4], fluorescence spectrophotometry [5], sensor methods [6], etc. HPLC and LC-MS are generally accepted as traditional methods, offering good detection accuracy and sensitivity. However, their limitations include high equipment costs and time-consuming preparation [7]. Meanwhile, ELISA technology possesses certain restrictions, such as restricted sensitivity and false positives from non-specific binding [8]. The limits of microbial detection technologies may stem from their reliance on long incubation times [9]. Moreover, although fluorescence spectrophotometry offers high sensitivity, its measurement precision is relatively poor, as evidenced by the substantial relative standard deviation (RSD) observed in repeated measurements [10]. In contrast, the sensor method offers a broad range of applications owing to its uncomplicated operation process, rapid analysis speed, excellent reproducibility, low cost, and high sensitivity [11].

To address the practical demand for effective monitoring of ENR residues, this study proposes for the first time a novel electrochemical sensor based on N/S CQDs@Fe-TCPP composite nanozymes. Compared to some current sensor methods for detecting ENR residues in food products, such as the fluorescence sensor method, the colorimetric sensor method, the microbial sensor method, and others [12], the electrochemical sensor method stands out due to its unique electrical signal response mechanism, not only offering highly efficient detection processes but also exhibiting exceptional sensitivity [13], and it has found widespread application in the detection of small molecules like antibiotics. Furthermore, by modifying the electrode surface with high-performance materials, electrochemical sensors can achieve significant improvements in sensitivity and selectivity. At present, electrochemical sensors have also found widespread application, particularly in the commercial sector. Nanozymes, as synthetic enzymes, are cost-effective and stable, often exhibiting favorable conductivity and catalytic properties, making them frequently employed as electrode modification materials [14]. For example, carbon quantum dots (CQDs), a class of conventional carbon-based nanozymes, exhibit significant potential in the construction of sensors due to their unique physicochemical properties [15]. Enhancing detection signals in analytical detection primarily hinges on utilizing the catalytic actions of nanozymes, whose activity can be boosted by regulating their electronic structure. So, an effective approach to enhancing catalytic efficiency is heteroatom doping technology. N and S doping can further enhance oxidation performance, improve electron–hole pair separation efficiency, and boost enzyme-like activity [16]. This demonstrates that N/S CQDs possess considerable catalytic application potential in the field of sensors. Despite the aforementioned advantages, N/S CQDs still undergo agglomeration during electrochemical detection, resulting in a substantial reduction in their catalytic efficiency. This severely limits their practical application. To this end, researchers have developed numerous CQD-based nanocomposites that aim to suppress CQDs agglomeration by constructing stable composite structures, thereby synergistically enhancing their performance, primarily encompassing metal-CQDs, polymer-CQDs, metal–organic frameworks MOFs-CQDs, and CQD-graphene composites [17]. Among the above many materials, MOFs demonstrate significant potential as ideal carriers because of their high porosity and enormous specific surface area [18]. They can immobilize and disperse CQDs within their pores and on their surfaces, thereby effectively mitigating agglomeration. Concurrently, MOFs exhibit outstanding catalytic performance owing to the dispersed active centers [19]. Fe-TCPP is an iron-based MOFs and there are a large number of catalytically active sites inside its structure. In the presence of hydrogen peroxide, Fe^2+^ and Fe^3+^ ions undergo the Fenton reaction to generate hydroxyl radicals (·OH), exhibiting outstanding peroxidase activity [20]. Fe-TCPP can act as nanoreactors to confine guest species, which often leads to synergistic effects and enhanced stability [21,22]. In this work, by uniformly dispersing N/S CQDs within the structure of Fe-TCPP, we aimed to achieve a pronounced synergistic effect. This strategy not only effectively immobilizes the N/S CQDs but also significantly enhances the electron–hole separation efficiency of the host framework, thereby achieving a synergistic enhancement of catalytic performance. However, no literature has yet reported the dedicated use of this synergistic N/S CQDs@Fe-TCPP composite for the direct electrochemical detection of ENR, indicating a gap in the development of simplified, highly sensitive sensors for this critical antibiotic.

In this investigation, composite nanozyme with excellent peroxidase-like activity were successfully fabricated. First, the N/S CQDs@Fe-TCPP were synthesized via a one-pot hydrothermal process through the synergistic assembly of Fe^3+^, tetrakis (4-carboxyphenyl) porphyrin (TCPP), and pre-synthesized N/S CQDs. During this process, the Fe-TCPP complex formed in situ and was simultaneously anchored onto the N/S CQDs surface. Then, the prepared N/S CQDs@Fe-TCPP were applied on bare electrodes using a simple drop-coating method. The nanozyme formed by complexing heteroatom-doped CQDs with Fe-TCPP combine the unique properties of both, exhibit more excellent catalytic efficacy, and significantly enhance their peroxidase-like activities. We optimized the key experimental parameters that could influence the electrochemical sensor’s performance, including the ratio of Fe and tetrakis TCPP, DL-malic acid, L-alanine, L-cysteine molar ratios, dialysis time, N/S CQDs added quality, modified electrode drop coating amount, buffer solution pH, H_2_O_2_ concentration, and volume of H_2_O_2_ addition. In addition, the suitability of the sensor in animal-derived food products like milk was investigated. Due to the faint signal of trace analytes being difficult to detect directly, indirect detection methods have been developed. These achieve quantification by detecting the probe signal affected by the analyte. In contrast to indirect detection methods, our selection of high-performance materials and continuous optimization of material ratios maximize material performance and sensitivity, enabling even minute concentrations to be converted into effective detection signals with a higher detection limit. Moreover, it not only surpasses the detection limits of most conventional direct methods in performance but also achieves sensitivity similar to some complex indirect detection methods [23,24,25]. And to our knowledge, this represents the first report of utilizing N/S CQDs@Fe-TCPP composite nanozyme-modified electrodes to establish a novel direct electrochemical sensing method for ENR. This approach offers straightforward operation and high sensitivity, presenting a highly promising new pathway for the direct detection of ENR residues in animal-derived foods via electrochemical sensors, which holds great potential for practical food safety applications.

## 2. Materials and Methods

### 2.1. Apparatus and Reagents

Apparatus: The external morphology and microstructure of N/S CQDs and N/S CQDs@Fe-TCPP were analyzed by scanning electron microscope (SEM) (ZEISS Sigma 360, Germany). An Ultima IV X-ray diffractometer (XRD) (Rigaku Ultima IV, Saitama, Japan) was used for phase identification. The molecular structures and functional groups were characterized by Fourier transform infrared spectroscopy (FTIR) (Thermo Fisher Scientific Nicolet iS20, Waltham, MA, USA). The successful synthesis of Fe-TCPP and the composite N/S CQDs@Fe-TCPP, as well as the efficient integration of heteroatoms, was confirmed using Energy Dispersive Spectroscopy (EDS) (ZEISS Sigma 360, Munich, Germany) mapping. The method employed for standard method comparison was high-performance liquid chromatography (HPLC) (Shimadzu LC-40, Kyoto, Japan). Electrochemical measurements were conducted using a three-electrode electrochemical workstation (CHI660E, Shanghai, China). In the conventional three-electrode setup, a glass carbon electrode (GCE, 3.0 mm in diameter) served as the working electrode, a calomel electrode (3 M KCl) served as the reference electrode, and platinum wire served as the counter electrode. All electrochemical investigations were carried out at room temperature.

Reagents: The antibiotics, including enrofloxacin (ENR, >98%), norfloxacin (NOR, >98%), ciprofloxacin (CIP, >98%), danofloxacin (DAF, >98%), thiamphenicol (THI, >98%), cephalosporins (CEF, >98%), and tetracycline (TCY, >98%) were supplied by Shanghai Yuanye Biotechnology Co. (Shanghai, China). Milk samples were purchased from local markets (Ya’an, China). S1 of the Supplemental Material contains a detailed list of the other reagents used.

### 2.2. Preparation of N/S CQDs

Referring to J.-W. Kang et al. [26], a hydrothermal technique was used to prepare N/S CQDs. Briefly described as follows: 1.34 g of DL-malic acid, 0.872 g of L-alanine, and 0.024 g of L-cysteine were weighed and mixed with 10 mL of ultrapure water, transferred to a microwave oven, and reacted on high at maximum power for 5 min. After the reaction was finished, it was cooled to room temperature, 10 mL of ultrapure water were added, and a water bath at 70 °C was employed for 30 min, and the resultant mixture was centrifuged for 20 min at 10,000 revolutions per minute, followed by filtration using a needle filter, and then dialyzed for several days in ultrapure water using a dialysis bag with a 1000 Da molecular weight cut-off. After dialysis, the N/S CQDs were lyophilized at −80 °C for 48 h to obtain brown colored carbon quantum dot powder.

### 2.3. Preparation of N/S CQDs@Fe-TCPP

With a few modest adjustments [27], N/S CQDs@Fe-TCPP were created. First, two different dispersion liquids were sonicated: dispersion liquid A, which contained 4.0 mg of Fe(NO_3_)_3_·9H_2_O, 10 mg of polyvinylpyrrplidone (PVP), 40 μL of CF_3_COOH, 9 mL of N,N-dimethylformamide (DMF), and 3 mL of C_2_H_5_OH, and dispersion liquid B, which contained 4.4 mg of TCPP, 3 mL of DMF, and 1 mL of C_2_H_5_OH. Then, Liquid A was combined with Liquid B. N/S CQDs were added to the mixture and homogeneously blended using ultrasound. After a final transfer, the mixture was dried for 24 h at 90 °C in a polytetrafluoroethylene reactor. The resultant powders were exposed to three sequential pumping treatments of DMF, deionized water, and acetone, and then vacuum-dried for an entire night at 60 °C.

### 2.4. Preparation of N/S CQDs@Fe-TCPP/GCE

The GCE was polished using a 0.3 um alumina paste on a polishing cloth before modification. It was then ultrasonically cleaned for 5 min each in ethanol (95%) and ultrapure water, respectively. To obtain a reliable response, the bare electrode was routinely cycled between −0.2 V and 0.6 V using cyclic voltammetry (CV) at a scan rate of 50 mV/s. To prepare N/S CQDs@Fe-TCPP/GCE, 4 mg of N/S CQDs@Fe-TCPP and 1 mL of ultrapure water were mixed and sonicated until a homogeneous solution was obtained, and then the solution was applied onto bare glassy carbon electrodes by the drop-coating method (modification volume from 2 µL to 10 µL). The electrodes were then thoroughly dried in a vacuum oven. The modification was repeated 3 times.

### 2.5. Electrochemical Measurement and Investigation of Reaction Mechanisms

To elucidate the detection mechanism of ENR and systematically evaluate the electrochemical performance of the prepared materials, bare electrodes, N/S CQDs-modified electrodes, and N/S CQDs@Fe-TCPP composite-modified electrodes were sequentially immersed in 10 mL PBS buffer solutions containing an equal concentration of ENR solution. Current responses within the 0.4–1.3 V potential range were recorded using differential pulse voltammetry (DPV), with pulse parameters set as follows: pulse amplitude 0.05 V, pulse width 0.05 s, and pulse period 0.5 s. The sensor was immersed in the solution under test for 100 s. By comparing the peak currents at the characteristic potential of approximately +0.8 V across the fabricated electrodes, the electrocatalytic enhancement effect of the material on the electrodes was evaluated.

### 2.6. Optimisation of Experimental Conditions

The optimization of experimental conditions encompasses both material optimization and test condition optimization. Electrochemical measurements were performed using an electrochemical workstation (CHI 660E, Shanghai, China) equipped with three electrodes, consisting of a Pt wire electrode and a Ag/AgCl electrode as counter electrode and reference electrode, respectively, as well as a glassy carbon electrode as working electrode.

The detection method is as follows: the modified electrode is immersed in PBS buffer (pH 8.0) containing a fixed concentration of ENR and 20 mM H_2_O_2_, and measurements are performed using DPV. The DPV scan range is from +0.4 V to +1.3 V, within which an irreversible anodic peak associated with ENR oxidation is observed at approximately +0.8 V. Following each measurement, the background correction signal is obtained by subtracting the DPV curve of the blank buffer solution, which contains no ENR, from the DPV curve of the sample. Baseline correction was performed using Origin 2021 software. First, through single-factor experiments, the preparation conditions potentially affecting the performance of N/S CQDs@Fe-TCPP materials were optimized, including N/S CQDs added quality (5 mg, 10 mg, 15 mg, 20 mg), dialysis time (1 d, 2 d, 3 d), the ratio of Fe to TCPP (1:1, 2:1, 3:1), and DL-malic acid, L-alanine, and L-cysteine molar ratios (10:9.0:1.2, 10:9.8:0.2 and 10:5.0:5.0).

With the optimal material composition determined, the experimental conditions for ENR detection were subsequently optimized by monitoring the changes in the oxidation peak current at approximately +0.8 V. These included drop coating volume (2 µL, 4 µL, 6 µL, 8 µL, 10 µL), buffer solution pH (ranging from 4.0 to 8.0), H_2_O_2_ concentration (0 mM, 10 mM, 20 mM, 30 mM, 40 mM), and volume of H_2_O_2_ addition (50 µL, 100 µL, 150 µL, 200 µL, 250 µL).

For the optimization of relevant conditions, we employed the method of controlling variables: for instance, under the fixed condition of adding 100 µL of H_2_O_2_, we optimized its optimal concentration; subsequently, at the determined optimal concentration, we further optimized the addition volume of H_2_O_2_.

To ensure data reliability, all optimization experiments were conducted using at least three independently prepared modified electrodes, with each electrode undergoing three measurements under identical conditions.

### 2.7. Evaluation of the Detection Performance of Sensors

Selectivity, reproducibility, stability, anti-interference, and limit of detection (LOD) are all factors considered in the assessment of detection performance. To ascertain each sensor contribution to the signal change, DAF, CIP, NOR, THI, CEF, and TCY were selected in order to take selectivity into account. Furthermore, measurements employing six identical sensors from the same batch were utilized to assess the reproducibility of the manufactured sensors. To assess the stability of the sensor over a 21-day period, the built sensors were tested every 7 days. Subsequently, the anti-interference capability of sensors was next tested by adding Na^+^, K^+^, Cl^−^, Fe^2+^, SO_4_^2−^, scorbic acid, and glucose to the ENR standard solution at concentrations 100 times that of ENR. Systematic testing was conducted on ENR standard solutions of varying concentrations using the fabricated sensor, establishing a linear connection between peak current and ENR content. Then, the LOD for this method was calculated according to Equation (1), yielding a correlation coefficient R^2^.

(1)  LOD=3α/k 

where α is the standard deviation (I) of the six blank values, and K is the slope of the linear regression curve.

### 2.8. Sample Preparation and Methodological Comparison

Samples of milk were made with slight modifications [28]. Firstly, 5 g ± 0.05 g of milk was weighed and put in a centrifuge tube. Next, 10 mL of acetonitrile was added for extraction. Vortexing and mixing, shaking at medium speed for 2 min, and then sonication for 10 min. After that, the supernatant was centrifuged for 5 min at 10,000 rpm. The supernatant was taken in another centrifuge tube, and as described above, 10 mL of acetonitrile was added to the centrifuge tube again for a second time. The supernatants obtained from the two operations were combined and fixed with acetonitrile for subsequent testing. Spiked samples with 500 nmol/L ENR were diluted 10 and 100 times using unspiked matrix in order to evaluate matrix effects. All samples underwent a standardized pretreatment procedure and were stored at 4 °C for subsequent analysis. These diluted samples were employed in spiked recovery experiments, with the sensor’s accuracy and reliability assessed through recovery rate calculations. For methodological validation, milk samples were additionally processed according to the Chinese national standard GB 29692-2013 [29] with comparative analysis conducted using HPLC.

### 2.9. Data Processing and Analysis

Data were imported into Excel from the CHI660E system, and graphs were plotted by Origin 2023. The Duncan’s multiple range test was applied to analyze the data, and *p* < 0.05 was deemed significant. Experiments were based on multiple samples.

## 3. Results

### 3.1. Characterization of Materials and Polymers

**Figure 1:**
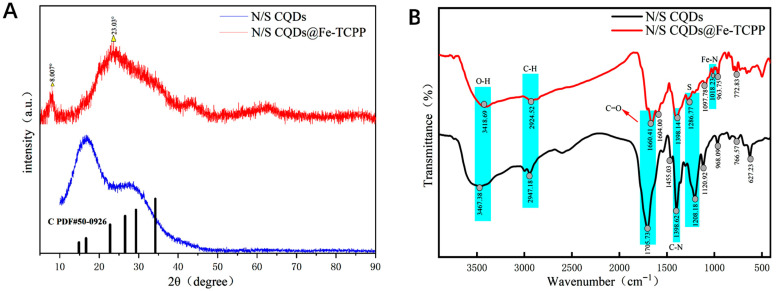
(**A**) XRD spectra of N/S CQDs and N/S CQDs@Fe-TCPP. (**B**) FTIR spectra of N/S CQDs, and N/S CQDs@Fe-TCPP.

The XRD patterns of N/S CQDs and N/S CQDs@Fe-TCPP are shown in Figure 1A. As shown, the N/S CQDs exhibit a pattern consistent with the standard spectrum, indicating successful crystalline phase integration. The diffraction pattern of N/S CQDs displays a broad, diffuse peak at 2θ ≈ 23°, a characteristic feature of CQDs This confirms their amorphous graphitic carbon structure dominated by short-range order and low crystallinity [30].

The XRD pattern of N/S CQDs@Fe-TCPP exhibits distinct structural changes. A sharp diffraction peak appears at 2θ = 8.0°, corresponding to one of the characteristic peaks of Fe-TCPP [31], confirming the successful incorporation of the Fe-TCPP crystalline phase into the composite. Simultaneously, the broad peak at 2θ = 23.03° persists, indicating that the carbon framework structure of N/S CQDs remains largely unchanged after composite formation.

Additionally, the full width at half maximum (FWHM) of the characteristic Fe-TCPP diffraction peak at 8.0° is relatively large, approaching 20°, reflecting the nanoscale grain size of Fe-TCPP in the composite material. Moreover, this pronounced peak broadening typically originates from microstrain and crystal defects within the lattice. Therefore, while the appearance of this characteristic peak confirms the formation of the Fe-TCPP phase, its broadened shape indicates that this phase consists of fine, imperfect grains rather than highly ordered bulk crystals.

This suggests that, although the crystalline integrity of Fe-TCPP in the composite is relatively limited, its nanocrystalline structure and associated lattice defects may provide abundant active sites. These structural features facilitate interfacial electron transfer and radical generation, significantly enhancing the material’s peroxidase-like catalytic activity.

Functional groups in the composite material were identified via FTIR, as shown in Figure 1B. The coordination between organic and metal ligands exhibited sharp peak shapes. A distinct broad hydroxyl (O-H) absorption band at 3467 cm^−1^ appeared in the N/S CQDs, indicating the presence of surface (O-H) groups and confirming that N/S CQDs are rich in surface O-H groups. A characteristic peak at 1705 cm^−1^ indicates the presence of carboxyl (C=O) groups, attributed to the stretching vibration of C=O bonds. Thus, FTIR results further confirm the existence of both O-H and C=O groups on the surface. Additionally, a prominent peak at 1398 cm^−1^ corresponds to C-N bond stretching vibrations, indicating successful introduction of -NH_2_ groups onto N/S CQDs [32]. The peak at 1208 cm^−1^ reveals the presence of sulfur functional groups [33], confirming the existence of sulfur functional groups within N/S carbon nanodots. In the FTIR spectrum of N/S CQDs@Fe-TCPP, a broad absorption band observed between 3700 and 3000 cm^−1^ corresponds to surface-adsorbed free amino (N-H) and O-H groups. Additionally, a peak at 1660 cm^−1^ indicates the presence of C=O groups, which remains attributable to C=O bond stretching vibrations. However, the peak intensity is reduced due to coordination interactions between the C=O groups and Fe^3+^. Concurrently, the absorption peak detected near 1018 cm^−1^ can be attributed to Fe-N vibrational modes, providing further spectroscopic evidence for the presence of Fe^3+^ [34] The broadening of the peak at 1286 cm^−1^ indicates that the introduction of Fe-TCPP reduces the crystallinity of the composite material. However, the spectrum of the N/S CQDs@Fe-TCPP nanocomposite is similar to that of N/S CQDs, exhibiting only peak broadening and slight shifts, suggesting the formation of hydrogen bonds and interactions between Fe-TCPP and N/S CQDs. The analysis confirms the fundamental structure of the N/S CQDs@Fe-TCPP composite, whose structural integrity remains preserved without alteration due to the successful loading of N/S CQDs.

**Figure 2:**
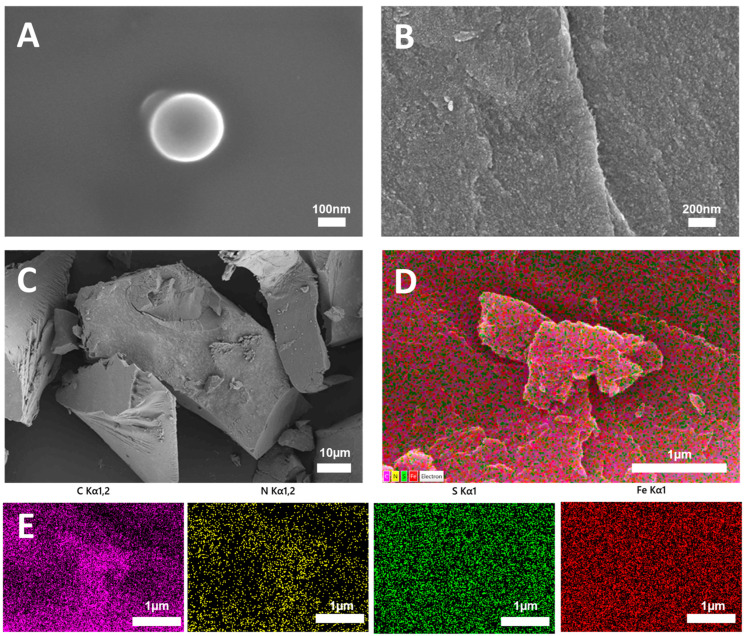
(**A**) SEM images of N/S CQDs. (**B**,**C**) SEM images of N/S CQDs@Fe-TCPP. (**D**) elemental mapping and (**E**) EDS of N/S CQDs@Fe-TCPP.

As shown in Figure 2A, under the ethanol ultrasonication process, N/S CQDs exhibited a typical spherical morphology with a smooth surface and excellent dispersion [35]. However, for N/S CQDs@Fe-TCPP, the morphology was altered. Figure 2B revealed a uniform particle distribution exhibiting large, regular-shaped particles with a rough surface. Figure 2C further demonstrates that the prepared nanocomposite exhibits an aggregated or densely packed blocky structure with wrinkled surfaces and irregular dimensions. This may be attributable to the non-uniform fragmentation of the nanocomposite resulting from the ultrasonic process employed during preparation [36]. The spatial elemental distribution and chemical makeup of the N/S CQDs@Fe-TCPP composite were investigated using the EDS mapping technique. Figure 2D,E demonstrate that C, N, S, and Fe were evenly dispersed throughout the composite material, thereby verifying the effective integration of N and S doping alongside the introduction of Fe.

### 3.2. Mechanism Exploration and Electrochemical Performance

Employing a heteroatom doping strategy, the N/S CQDs developed in this work feature surfaces rich in diverse functional groups. These provide abundant active sites capable of effectively anchoring Fe-TCPP through interactions such as coordination or hydrogen bonding, thereby promoting synergistic effects within the composite material [37,38]. Subsequently, N/S CQDs bind to Fe-TCPP through interactions between iron and amino groups. N/S CQDs@Fe-TCPP are applied onto the exposed GCE surface, enhancing the electrochemical reaction signal and improving detection sensitivity. The nanocatalyst with peroxidase activity prepared in this study significantly promotes the generation of ·OH in the presence of H_2_O_2_, with ·OH playing a pivotal role in the transformation of ENR. Specifically, as illustrated in Figure S1A, the aromatic skeleton of ENR undergoes hydroxylation, while the piperazine moiety achieves complete degradation primarily via radical coupling and hydrolysis pathways [39]. These transformation processes are both dependent on high concentrations of ·OH. That is, the oxidative degradation efficiency of ENR directly depended on the concentration of ·OH, with higher concentrations driving the reaction toward deeper oxidation. The electrochemical signal gets better as the ENR concentration rises, and the relationship between the oxidation peak current and various ENR concentrations is the basis for ENR detection.

The sensing performance of the modified electrodes was directly evaluated by comparing ENR oxidation peak currents obtained from DPV under identical conditions. By comparing the ENR peak currents of different electrodes (bare GCE, N/S CQDs/GCE, N/S CQDs@Fe-TCPP/GCE), their performance differences were obtained. As demonstrated in Figure S1B, the N/S CQDs/GCE have higher response currents than bare GCE. This phenomenon was attributed to the inherent excellent electrical conductivity of the N/S CQDs and the enhanced catalytic activity from N and S doping, which facilitated the electron transfer kinetics of ENR oxidation [40,41]. Due to the presence of highly distributed catalytically active sites in the MOFs backbone, N/S CQDs@Fe-TCPP/GCE exhibited a greater current response than N/S CQDs/GCE, which facilitated full contact between ENR and the catalytically active sites [42]. The high specific surface area of the composite also exposed more active sites, enabling fuller integration of N/S CQDs with Fe-TCPP and improved utilization of intrinsic active sites. This synergistic effect ultimately enhanced the catalytic ability of the composite, thereby accelerating the electrochemical oxidation of ENR [43].

### 3.3. Optimization of Experimental Conditions

#### 3.3.1. Optimization of Fabrication Conditions of N/S CQDs@Fe-TCPP/GCE

**Figure 3:**
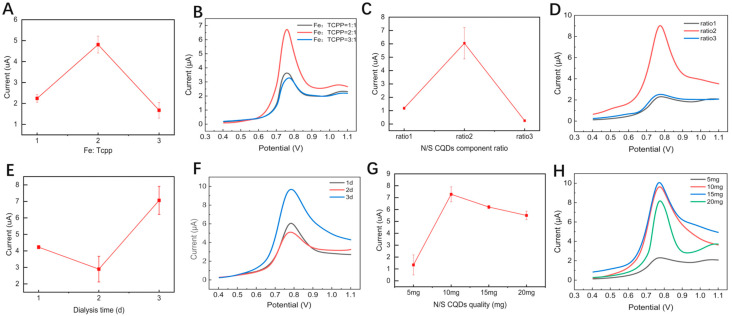
The relationships between (**A**,**B**) the ratio of Fe and TCPP, (**C**,**D**) the three synthetic ratios of DL-malic acid, L-alanine, and L-cysteine (10:9.0:1.2, 10:9.8:0.2 and 10:5.0:5.0), (**E**,**F**) optimization of dialysis time, and (**G**,**H**) N/S CQDs add quality on the peak current (I) measured by DPV in 0.2 mol/L PBS buffer solution.

The structure and properties of nano-enzymatic materials can change significantly depending on the preparation process and conditions. These variations are mainly affected by a variety of factors such as synthesis ratio, preparation temperature, heating rate, and gas atmosphere [44]. To increase the conductivity and improve the performance of N/S CQDs@Fe-TCPP/GCE, we optimized the composite ratio of Fe and TCPP, the DL-malic acid, L-alanine, and L-cysteine molar ratios, the dialysis time of CQDs, and the N/S CQDs add quality. The DPV technique (0.4–1.3 V, 50 mV/s) was employed as an indication to quantify the current differential between the electrodes before and after modification. Based on the findings of the experiment, when the composite ratio of Fe and TCPP was 2:1 (Figure 3A,B), the DL-malic acid, L-alanine, and L-cysteine molar ratios were 10:9.8:0.2 (Figure 3C,D), the dialysis time of CQDs was 3 days (Figure 3E,F), and the N/S CQDs added quality was 10 mg (Figure 3G,H), the prepared N/S CQDs@Fe-TCPP/GCE exhibited the highest current response. The data points represent the mean ± standard deviation of triplicate measurements (n = 3).

#### 3.3.2. Optimization of Detection Conditions of N/S CQDs@Fe-TCPP/GCE

Important experimental parameters, including drop coating volume, buffer solution pH, H_2_O_2_ concentration, and H_2_O_2_ addition, were optimized to achieve the conditions for highly sensitive ENR detection. The outcomes are displayed below.

**Figure 4:**
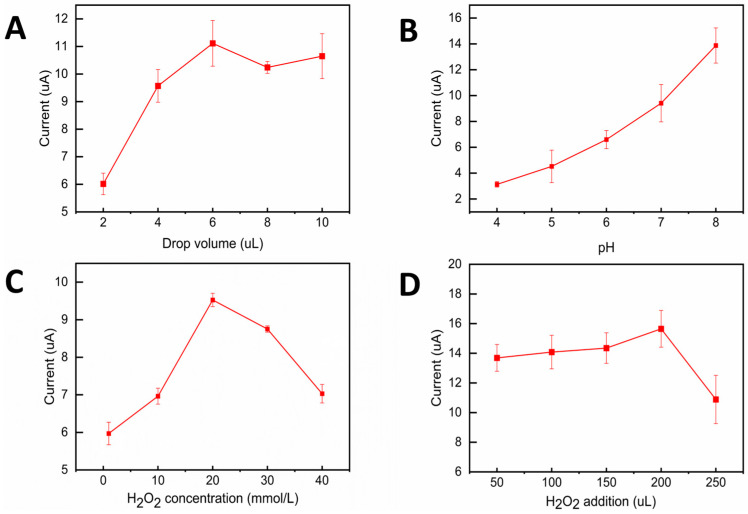
The plots for the relationship of (**A**) modified electrode drop coating amount, (**B**) background pH value of electrochemical reactions, (**C**) detection of the concentration of H_2_O_2_ added to the solution, and (**D**) detection of the volume of H_2_O_2_ addition added to the solution on the peak current (I) measured by DPV in 0.2 mol/L PBS buffer solution.

The drop coating volume plays a significant role in the detection of ENR. As shown in Figure 4A, the current response of ENR was optimal when the drop coating volume of N/S CQDs@Fe-TCPP was 6 μL. The current response of ENR gradually increased when the drop coating volume increased from 2 μL to 6 μL, but the response current showed a decreasing trend when the drop coating volume exceeded 6 μL. This is because, when the drop-coating volume is less than 6 μL, the amount of modification is insufficient, resulting in the material not being able to cover the electrode surface sufficiently, and the response current is low; when the drop-coating volume is greater than 6 μL, the modification material builds up on the surface of the electrode, which ultimately prevents the transport of electrons, lowering the response current. Therefore, 6 μL was chosen as the optimal drop coating volume for subsequent experiments. The interaction between N/S CQDs@Fe-TCPP and ENR, as well as the stability and activity of their peroxidase-like enzymes, are affected by the pH of the buffer solution. Consequently, the selection of an appropriate pH buffer solution is a critical factor governing the performance of the sensor. The effect of pH on ENR detection was explored by preparing assay solutions containing the same amount of ENR at different pH (4, 5, 6, 7, 8). As can be seen from Figure 4B, the current response of ENR was maximum at pH = 8. This result indicated that the pH was optimal for the interaction between N/S CQDs@Fe-TCPP and ENR, and the reason is presumed to be that the surface hydroxyl groups of the metal nanoparticles (Fe^2+^, Fe^3+^) are more likely to be deprotonated under slightly alkaline conditions to form more stable catalytically active sites, which accelerates the kinetic process of the reaction.

The assay accuracy and reaction rate were significantly impacted by the H_2_O_2_ concentration and addition volume. The reaction rate is strongly influenced by the concentration of H_2_O_2_ as one of the reaction substrates. The substrate inhibition effect, which prevents the activity of the nanozymes, can result from an excessively high H_2_O_2_ concentration. Even if the concentration is increased, the reaction rate tends to decrease. Conversely, if the concentration of H_2_O_2_ is too low, the reaction rate slows down, resulting in a weak current signal and low detection sensitivity. The addition of too little H_2_O_2_ produced too few reactive oxygen species, such as ·OH, resulting in ENR not being fully oxidized, generating insignificant changes in detectable signals, and affecting the quantitative analysis of ENR. Excessive addition volume of H_2_O_2_ will not only lead to changes in the volume of the reaction system, thus diluting other reaction components and possibly inducing other side reactions, but also will interfere with the detection results. As shown in Figure 4C, when 100 μL of H_2_O_2_ was added, the peak current corresponded to an H_2_O_2_ concentration of 20 mM. The data points represent the mean ± standard deviation of triplicate measurements (n = 3). Similarly, Figure 4D shows that when the H_2_O_2_ concentration was 20 mM, the peak current corresponded to an added volume of 200 μL of H_2_O_2_. Each measurement is repeated three times.

### 3.4. Detection Performance Evaluation of the N/S CQDs@Fe-TCPP/GCE Sensor

**Figure 5:**
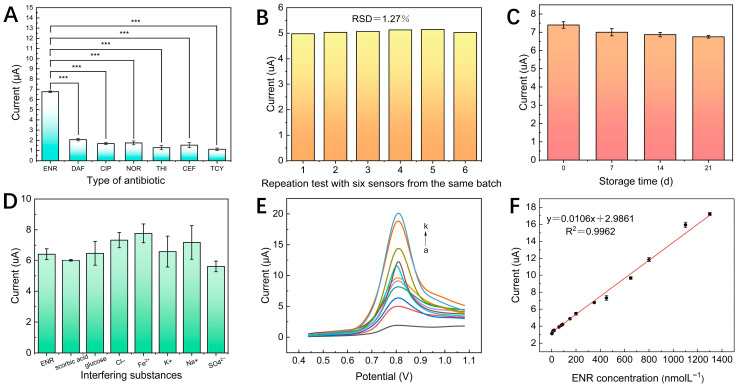
(**A**) Selectivity of the N/S CQDs@Fe-TCPP/GCE Sensor. *** indicates *p* < 0.001. (**B**) Reproducibility of the N/S CQDs@Fe-TCPP/GCE Sensor. (**C**) Stability of the N/S CQDs@Fe-TCPP/GCE Sensor. (**D**) Anti-interference of the N/S CQDs@Fe-TCPP/GCE Sensor. (**E**) In a 0.2 mol/L PBS buffer solution, N/S CQDs@ Fe-TCPP/GCE DPV curves were obtained with varying ENR concentrations added (0, 50, 100, 200, 300, 350, 450, 650, 800, 1100, and 1300 nM) (a → k). (**F**) The relationship between peak current and ENR concentration. Data points represent the mean ± standard deviation (n = 3).

To assess the selectivity of the prepared sensor, a certain concentration of ENR solution was quantitatively analyzed with other antibiotics, including DAF, CIP, and NOR, which are structurally similar to ENR, as well as THI, CEF, and TCY, which are structurally different from ENR. The concentration of ENR was fixed at 300 nM. For comparison, the test concentration of its structural analogue was set at 30 µM, representing 100 times the ENR concentration, whereas the test concentration of the non-structural analogue was set at 15 µM, equivalent to 50 times the ENR concentration. As seen in Figure 5A, the sensor showed a minor current response for the structure analogs, which was marginally higher than that for the non-structural analogs, and the strongest current response for ENR among other antibiotics at concentrations significantly higher than ENR. According to the findings, the sensor exhibits strong ENR identification specificity and selectivity.

By creating six identical N/S CQDs@Fe-TCPP/GCE sensors from the same batch and utilizing standard solutions of ENR at particular concentrations as controls, the reproducibility of the sensor was investigated. As shown in Figure 5B, there was a certain fluctuation between the results determined by six identical sensors, and the RSD of the ENR determination was 1.27%. This result revealed that the N/S CQDs@Fe-TCPP/GCE sensor possesses satisfactory reproducibility.

In order to investigate the stability of the N/S CQDs@Fe-TCPP/GCE sensors, the prepared sensors were stored at 4 °C for 21 days, during which a set of sensors was taken out every 7 days and electrochemically detected against the equal concentration of ENR, and the peak currents were recorded using the DPV method. The results of the stability tests are shown in Figure 5C. Several observations of Figure 5C showed that as the storage period rose, the peak current of the modified electrode utilized to detect ENR gradually reduced. The response current of ENR decreased on the 14th and 21st days of storage. This phenomenon may be attributed to the oxidation of the electrode surface modification materials over time, which in turn leads to a continuous weakening of the current response [45]. However, at 14 d and 21 d, the response currents of the ENR were still 93.51% and 91.89% of the initial values, respectively, indicating the excellent stability of the prepared N/S CQDs@Fe-TCPP/GCE sensor.

Common ingredients in food, such as metal ions, carbohydrates, and vitamins, were selected for the anti-interference study of the sensors. It was determined that the additional chemicals had no discernible impact on the test samples when the ΔI value was less than 5% [46]. The results are shown in Figure 5D. When the ENR concentration was 300 nM, it was observed that the addition of Cl^−^, Na^+^, K^+^, SO_4_^2−^, Fe^2+^, glucose, and scorbic acid at high concentration multiples did not produce significant changes in the ENR currents, and the relative errors were all less than ±4.9%. Excellent selectivity, reproducibility, stability, and anti-interference are confirmed by thorough evaluation metrics, fulfilling all necessary requirements for practical electrochemical sensor applications.

To confirm the effectiveness of the constructed sensors, N/S CQDs@Fe-TCPP/GCE sensors were tested under optimal experimental conditions for solutions containing different concentrations of ENR (1, 10, 20, 60, 80, 100, 150, 350, 450, 650, 800, 1100, and 1300 nM). Figure 5E showed that the peak current increases with increasing ENR concentration. At the same time, Figure 5F further indicated a clear linear relationship between the peak current and ENR concentration, and the corresponding linear equation is I (µA) = 0.0106c (nM) + 2.9861 (R^2^ = 0.9962), where I is the peak current after the sensor adsorbs ENR, and c stands for the concentration of the ENR standard solution. The LOD for ENR was calculated to be approximately 0.872 nM based on the equation 3α/K, where α is the standard deviation (I) of the eleven blank values (when the ENR concentration approaches the blank value, there is still a current response) and K is the slope of the linear regression curve.

### 3.5. Application of Real Samples Detection

In view of the fact that milk is prone to contain ENR residues in the actual production process, we chose milk as the test sample to determine whether the sensor can be used in practical applications and validated it by HPLC. Milk samples were purchased from Ya’an Jixuan Supermarket. Following basic treatment, three parallel measurements of the spiked samples at three spiked levels (n = 3) were made to evaluate the sensor’s accuracy. The specificity of the sensor was confirmed by comparing the responses of unspiked and ENR-spiked milk samples. The blank milk sample showed no oxidation peak at the characteristic potential of ENR (approximately +0.8 V). Upon the addition of ENR, a single, well-defined oxidation peak appeared at this potential, with no additional peaks observed, demonstrating the sensor’s high specificity for ENR detection. Based on these specific signals, the recoveries were computed. As indicated in Table 1, the recoveries of ENR in milk samples using the N/S CQDs@Fe-TCPP/GCE varied from 99.02% to 100.9% with RSD ≤ 4.80%, which was superior to HPLC and extremely consistent with it, ranging from 72.2% to 93.42%. The research findings indicate that no ENR residues were detected in the commercially available milk samples within the scope of this study. We found that when the ENR concentration was 5 nM, the recovery rate obtained by HPLC was only 72.2%. In contrast, the recovery rate obtained by our experimental method was significantly closer to 100%, indicating that the results obtained were superior. We speculated that the reason for this may be that when detecting trace amounts of ENR, the HPLC method involves numerous steps and is more prone to losses during sample pretreatment, leading to deviations in the results. The sensor prepared in this experiment has high sensitivity, strong anti-interference ability, and a simple sample pretreatment method, resulting in more accurate detection results. When detecting higher ENR concentrations, the results obtained by this experimental method were basically consistent with those obtained by HPLC. These results fully proved that the developed sensor has high applicability in detecting ENR in actual samples.

Table 2 presents a comparison between the sensor developed in this study and previously reported methods for ENR detection, highlighting the advantages of the proposed approach. Firstly, The suggested sensor has a low LOD of 0.872 nM in terms of detection sensitivity. Compared to electrochemical sensors employing direct ENR detection, its LOD was lower by 88.66–99.97% relative to values listed in the table, and its performance was comparable to various indirect ENR detection methods [47,48]. Secondly, compared to indirect detection methods typically requiring multi-step synthesis and complex modifications—such as the dual-mode colourimetric aptasensor based on Au NRs/PDA-LFA developed by Chen et al. [49]. Their methods are highly accurate, but cumbersome to implement. In contrast, this study employs a one-pot hydrothermal method to prepare the core composite material, significantly simplifying the synthesis pathway for functional materials. Moreover, the entire detection process is intuitive to operate, with a single measurement completed in approximately 100 s, requiring minimal instrumentation and operator skill. Finally, regarding practical reliability, as demonstrated in Table 1 of the main text, this method exhibits ideal recovery rates (99.02% to 100.9%) for spiked milk samples. Furthermore, the RSD for parallel experiments consistently falls below 4.80%, confirming the accuracy and repeatability of results in complex real-world sample matrices. In summary, this demonstrates that the sensor material prepared in this study exhibits outstanding performance, significantly enhancing the sensitivity of ENR detection while effectively lowering the LOD. This bolsters the technology’s potential for future applications in complex real-world detection tasks, such as developing handheld detectors. This will also form one of our future research directions. This study selected milk, a complex matrix, as a representative substrate to validate the ENR detection method. However, the applicability of the conclusions to other food systems remains to be verified. Furthermore, whilst the experiments were conducted under controlled conditions, the long-term stability of the method and its performance in real-world complex food environments require confirmation through subsequent field studies.

## 4. Conclusions

An electrochemical sensor integrating direct detection with straightforward operation has fulfilled the requirement for rapid and precise detection of ENR. It was confirmed that the synergistic effect of N/S CQDs@Fe-TCPP collectively enhanced the exposure degree and utilization efficiency of active sites, as well as its enzyme-mimicking catalytic activity. In the range of 1–1300 nM of ENR, the electrochemical sensor that was created exhibited an LOD of 0.872 nM (S/N = 3) under ideal experimental circumstances. In addition, the sensor showed good selectivity, reproducibility, stability, and anti-interference, and it was successfully applied to the detection of ENR residues in milk samples. The recoveries of the samples ranged between 99.02% and 100.9% with RSD ≤ 4.80%. In summary, the complementary integration of N/S CQDs and Fe-TCPP renders them ideal materials for sensor construction. Compared to traditional analytical methods, the rapid and convenient nature of this electrochemical sensor confers significant advantages in specific scenarios, such as on-site testing, for instance, in the development of handheld detectors. In future iterations, priority improvements could focus on enhancing material selectivity through molecular imprinting techniques while integrating artificial intelligence to further boost analytical efficiency. Concurrently, the pursuit of advanced catalytic materials will propel sensing capabilities to unprecedented heights.

## Figures and Tables

**Table 1:** N/S CQDs@Fe-TCPP/GCE and HPLC determination of ENR in actual food samples (n = 3).

Samples	Added (nM)	Found (nM)	Recovery (%)	RSD (%)	Found (nM)	Recovery (%)
		**this study**			**HPLC**	
Milk-1	0	nd ^a^	-	-		
Milk-2	5	5.04 ± 0.33	100.9 ± 0.07	4.48	3.61	72.2
Milk-3	50	49.51 ± 1.06	99.02 ± 0.02	4.80	44.50	89.0
Milk-4	500	498.84 ± 3.31	99.76 ± 0.01	0.34	467.10	93.42

nd ^a^ means not detected.

**Table 2:** Comparison of ENR detection sensors.

Methods	Technique	Samples	Linear Range (μM)	Limit of Detection (μM)	Ref.
AgInS2 QDs	Fluorimetry	milk	0.87–55.65	0.067	[50]
ENR-Nb66-vHRP	ELISA	milk and pork	0.03–0.68	0.018	[51]
COFs/AuNPs/GCE	Electrochemical	milk and water	0.05–10/ 10–120	0.041	[52]
Bare GCE	Electrochemical	water	1–30/ 30–100	31.0 ± 4.6 1.64 ± 0.3	[53]
N CQDs	Fluorimetry	water	2.78–41.74	0.45	[54]
AuNRs/PDA-LFA	Colorimetric	milk	0.00139–0.278	8.35 × 10^−4^ 3.34 × 10^−4^	[49]
N/S CQDs@Fe-TCPP	Electrochemical	milk	0.0001–1.3	0.00736	this work

## Data Availability

The original contributions presented in this study are included in the article/Supplementary Material. Further inquiries can be directed to the corresponding author.

## Supplementary Materials

The following supporting information can be downloaded at: https://www.mdpi.com/article/10.3390/foods15020266/s1, S1. Apparatus and reagents. Figure S1: (A) Oxidation pathways of ENR. (B) DPV curves at 20 mmol/L H_2_O_2_ concentration: bare GCE (a), N/S CQDs/GCE (b), and N/S CQDs@Fe MOFs/GCE (c).
