# Supplementary material for: Construction of N/S CQDs@Fe-TCPP Nanocatalyst-Induced Electrochemical Sensors for Rapid and Sensitive Detection of Enrofloxacin Residues in Milk
Source: Foods. 2026 Jan 11;15(2):266. doi: 10.3390/foods15020266 (PMC12840261; doi:10.3390/foods15020266)
Supplement: Supplementary file 1 [file foods-15-00266-s001.zip › foods-4061249-supplementary.pdf]

Supporting Information

Construction of an N/S CQDs@Fe-TCPP nanocatalyst-induced electrochemical sensors for rapid and sensitive detection of enrofloxacin residues in milk

Wenjing Wang[1,†],Shujuan Chen[1,†,*],Yifan Fu[1], Yike Hong[1], Chenbo Tang[1], Likou Zou[2], Junni Tang[3], Li He[1], Shuliang Liu[1], Kaidi Hu[1], Aiping Liu[1]

[1] College of Food Science, Sichuan Agricultural University, Ya'an, Sichuan 625000, PR China
[2] College of Resources, Sichuan Agricultural University, Chengdu, Sichuan 611130, PR China
[3]College of Pharmacy and Food, Southwest Minzu University, Chengdu, Sichuan 610225, PR China
[*]Correspondence: chenshujuan1@163.com; Tel:13688074131
[†]These authors contributed equally to this work

S1 Apparatus and reagents

DL-malic acid, L-alanine, L-cysteine, Polyvinylpyrrplidone(PVP), Trifluoroacetic acid, Nitric acid, sodium carbonate, ethanol, Ferric chloride hexahydrate ($FeCl_3 \cdot 6H_2O$), N,N-dimethylformamide(DMF) and other reagents were purchased from Chengdu Kelon Chemicals Co. H_2O_2, purchased from Shanghai McLean Biochemical Technology Co. Ultrapure water with resistivity over 18.25 $M\Omega\text{-}cm^{-1}$ was used in this study. All other compounds used in the experiment were of analytically grade.

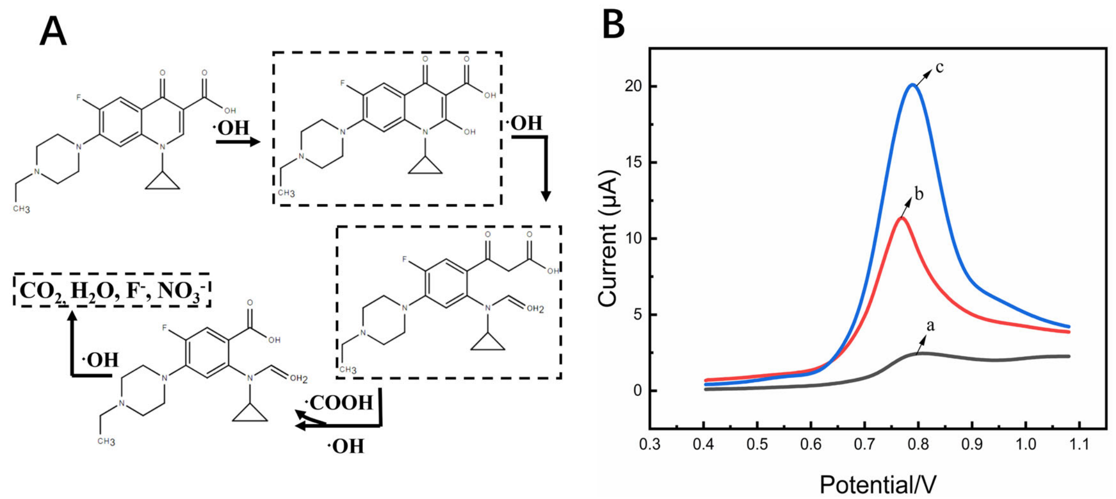

Figure. S1. (**A**) Oxidation pathways of ENR. (**B**) DPV curves at 20 mmol/L H_2O_2 concentration: bare GCE (a), N/S CQDs/GCE (b), and N/S CQDs@Fe MOFs/GCE (c).
